# Three-Dimensional Ultrasonic Needle Tip Tracking with a Fiber-Optic Ultrasound Receiver

**DOI:** 10.3791/57207

**Published:** 2018-08-21

**Authors:** Wenfeng Xia, Simeon J. West, Malcolm C. Finlay, Rosalind Pratt, Sunish Mathews, Jean-Martial Mari, Sebastien Ourselin, Anna L. David, Adrien E. Desjardins

**Affiliations:** ^1^Wellcome/EPSRC Centre for Interventional and Surgical Sciences, University College London; ^2^Department of Medical Physics and Biomedical Engineering, University College London; ^3^Department of Anaesthesia, University College Hospital; ^4^St Bartholomew's Hospital and Queen Mary University of London; ^5^Institute for Women's Health, University College London; ^6^Centre for Medical Imaging Computing, University College London; ^7^GePaSud, University of French Polynesia; ^8^Department of Development and Regeneration, KU Leuven (Katholieke Universiteit); ^9^NIHR University College London Hospitals Biomedical Research Centre

**Keywords:** This Month in JoVE, Issue 138, Ultrasonic tracking, ultrasound imaging, coded excitation, fiber-optic hydrophone, minimally invasive surgery, image reconstruction

## Abstract

Ultrasound is frequently used for guiding minimally invasive procedures, but visualizing medical devices is often challenging with this imaging modality. When visualization is lost, the medical device can cause trauma to critical tissue structures. Here, a method to track the needle tip during ultrasound image-guided procedures is presented. This method involves the use of a fiber-optic ultrasound receiver that is affixed within the cannula of a medical needle to communicate ultrasonically with the external ultrasound probe. This custom probe comprises a central transducer element array and side element arrays. In addition to conventional two-dimensional (2D) B-mode ultrasound imaging provided by the central array, three-dimensional (3D) needle tip tracking is provided by the side arrays. For B-mode ultrasound imaging, a standard transmit-receive sequence with electronic beamforming is performed. For ultrasonic tracking, Golay-coded ultrasound transmissions from the 4 side arrays are received by the hydrophone sensor, and subsequently the received signals are decoded to identify the needle tip's spatial location with respect to the ultrasound imaging probe. As a preliminary validation of this method, insertions of the needle/hydrophone pair were performed in clinically realistic contexts. This novel ultrasound imaging/tracking method is compatible with current clinical workflow, and it provides reliable device tracking during in-plane and out-of-plane needle insertions.

**Figure:**
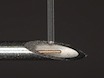


## Introduction

Accurate and efficient localization of invasive medical devices is highly desired in many ultrasound-guided minimally invasive procedures. These procedures are encountered in clinical contexts such as regional anesthesia and interventional pain management^1^, interventional oncology^2^, and fetal medicine^3^. Visualization of the medical device tip can be challenging with ultrasound imaging. During in-plane insertions, needles often have poor visibility when insertion angles are steep. Moreover, during out-of-plane insertions, the needle shaft can be misinterpreted as the needle tip. When the needle tip is not ultrasonically visible, it can cause complications by damaging critical tissue structures.

Many methods are available to localize medical devices during ultrasound imaging, but a reliable one that is compatible with current clinical workflow is highly desired. Echogenic surfaces can be used to improve visibility during steep angle in-plane insertions^4^. Electromagnetic tracking systems can be used during out-of-plane insertions, but electromagnetic field disturbances can severely degrade their accuracy. 3D ultrasound imaging can improve visibility of medical devices in certain cardiac and fetal procedures when they are surrounded by fluids^5^. However, 3D ultrasound imaging is not widely used for needle guidance, in part due to the complexities associated with image interpretation.

Ultrasonic tracking is a method that has shown great potential for improving medical device visibility^6^,^7^,^8^,^9^,^10^,^11^,^12^,^13^,^14^. With ultrasonic tracking, the medical device has an embedded ultrasound sensor or transmitter that actively communicates with the external ultrasound imaging probe. The medical device position can be identified from the measured ultrasound time-of-flights between the embedded ultrasound sensor/transmitter and different transducer elements of the probe. To date, ultrasonic tracking has been limited to in-plane tracking, which has greatly restricted its clinical use.

Here, a demonstration of how 3D ultrasonic tracking can be performed with a custom ultrasound imaging probe and a fiber-optic hydrophone affixed within the cannula of a needle is provided (Figure 1). This custom probe, which was designed by the authors and manufactured externally, comprises a central array of transducer elements and four side arrays. The central array is used for 2D ultrasound imaging; the side arrays, for 3D needle tip tracking in concert with the fiber-optic ultrasound receiver. It is shown how the fiber-optic ultrasound receiver can be positioned and affixed within the needle cannula, how the tracking accuracy of the system can be measured on the benchtop, and how clinical validation can be performed.

## Protocol

### 1. System Hardware

1. The clinical custom ultrasound imaging probe
  1. Create a draft design for the layout of the transducer elements in the custom probe that includes central and side arrays. Submit the design to the manufacturer of this probe.
  2. With feedback from the manufacturer, create a detailed design for the custom probe that includes refinements to the transducer frequency characteristics and geometries (Figure 2). NOTE: Typically, the manufacturer of the custom probe can design the electronic systems, the probe housing, and the probe connector for compatibility for a particular type of ultrasound imaging system. The manufacturer can also include an operation mode switch (hardware) to determine which set of 128 elements was addressed by the ultrasound imaging system. In imaging mode, the central array is addressed; in tracking mode, the side arrays are addressed.
2. The tracking needle
  1. Select a fiber-optic ultrasound hydrophone which comprises a single-mode optical fiber with a Fabry-Pérot cavity at the distal end (outer diameter (OD): 150 µm). NOTE: Hydrophones that comprise a single-mode optical fiber with a Fabry-Pérot cavity at the distal end (OD: 150 µm), are available commercially. Proximal to the distal end, optical fibers that are frequently used for telecommunications have a cladding layer (OD: 125 µm), a buffer layer (OD: 250 µm), and a jacket (OD: 900 µm).
  2. Using a scalpel, partially remove the 900 micrometer jacket along the length of the fiber optic hydrophone, close to its distal end, to expose the buffer layer until the hydrophone can fit within the needle cannula. NOTE: For mechanical robustness, it is useful to retain the protective buffer layer/jacket on the section of the fiber optic cable that is proximal to the Luer connector. Take care with handling the fragile section of the fiber after the jacket is removed, before it is protected by the needle cannula.
  3. Affix the medical needle horizontally to a manual horizontal translation stage, and visualize the needle tip with a stereo microscope, with the optical axis of the microscope aligned horizontally and perpendicular to the needle. If necessary, rotate the needle about its axis so that the bevel surface of the needle can be seen with the microscope.
  4. With the distal end of the needle in view of the microscope, insert the fiber-optic ultrasound receiver through the cannula of a Tuohy-Borst Sidearm adapter and subsequently through the Luer connector of the needle until the sensing region of the hydrophone is just proximal to the bevel surface of the needle. At this stage, the Sidearm adapter should not be connected to the needle. Affix the hydrophone to the translation stage (polyimide tape works well) to avoid its movement within the needle.
  5. Affix the hydrophone to the translation stage with polyimide tape to avoid movement of the device within the needle.
  6. Vertically affix a 20-microliter pipette to the vertical translation stage with the tip facing downward and use both the horizontal and vertical translation stages to position the micropipette tip until it is adjacent to the fiber-optic hydrophone and about 0.5 mm proximal to the sensing region at the distal end.
  7. Place a drop of optical adhesive at the proximal end of the micropipette and adjust the needle to allow a direct path from the micropipette tip to the fiber-optic ultrasound receiver.
  8. Then use a 10-mL syringe to apply pressure at the proximal end of the micropipette to gradually dispense the adhesive from the distal into the fiber-optic ultrasound receiver, taking care to avoid applying adhesive to the sensing region or occluding the cannula, and illuminate the needle tip with ultraviolet light until the optical adhesive is cured.

### 2. System Integration

1. Connect the hydrophone to its optical console. NOTE: Optical consoles that provide an analog voltage signal proportional to the received pressure are available commercially.
2. Connect the custom ultrasound imaging probe to the ultrasound console.
3. Perform interleaved acquisitions of B-mode ultrasound images and coded ultrasound pulses for tracking^10^,^14^. For B-mode ultrasound image acquisition, perform pulse-echo transmit-receive sequences with the central array elements. Use the hardware switch to control whether the side array elements or the central array elements are accessed.
4. Digitize the hydrophone signals and the timing signals according to the starts of ultrasound transmissions simultaneously with a data acquisition (DAQ) card.
5. Process and display the signals acquired from the pulse-echo transmit-receive sequences, to obtain B-mode ultrasound images. Additionally, process and display the hydrophone signals to localize the fiber-optic ultrasound receiver relative to the custom probe. For the latter task, the algorithms are described by Xia *et al*.^12^,^14^
6. Overlay the needle tip locations onto the B-mode ultrasound images. To display 3D tracking information onto a 2D ultrasound image display, the position of the needle tip (lateral and depth coordinates) can be indicated with a cross; the out-of-plane distance and side of the imaging plane, with the size and color of this cross, respectively.

### 3. Pre-clinical Validation

1. Select the operation mode using the switch on the ultrasound imaging probe.
2. Add ultrasound gel to the custom ultrasound imaging probe.
3. Prepare a fetal ultrasound phantom by adding water to mimic amniotic fluid.
4. Using the B-mode ultrasound imaging, identify the amniotic fluid in the phantom as the insertion target. NOTE: The insertion target will depend on the context; it could include a particular region of tissue for diagnosis or therapy during a clinical procedure, or a designated location in an imaging phantom to mimic a tissue region.
5. Insert the needle toward the insertion target. During the insertion, alternate between operation modes (imaging and tracking) continuously using the switch on the custom probe.

## Representative Results

The animal experiment was conducted in accordance with UK Home Office regulations and the Guidance for the Operation of Animals (Scientific Procedures) Act (1986). The sheep was housed in accordance with UK Home Office guidelines relating to animal welfare; the experiments were conducted under the Home Office Project License 70/7408 entitled "Prenatal therapy with stem cells and gene transfer". Ethics approval for sheep experiments was provided by the University College London, United Kingdom and the Animal Welfare Ethics Review Boards of the Royal Veterinary College.

With ethics approval in place, a pregnant sheep for preclinical *in vivo* validation was used. After receiving intravaginal progesterone suppositories for 2 weeks, ewes were time-mated to induce ovulation, as described by David *et al*.^34^ At 130 days of gestation, one pregnant ewe was starved overnight with a pregnant companion ewe. The ewe then underwent general anesthesia induced with thiopental sodium 20 mg kg^-1^ intravenously and was maintained with 2-2.5% isoflurane in oxygen after intubation via a ventilator. Correct intubation was confirmed by listening to the lungs bilaterally. Anesthesia was confirmed by assessment of the corneal reflex. Oxygen saturation was measured continuously using a saturation monitor on the tongue or ear. The ewe was placed on her back in semi-recundancy and a nasogastric tube was passed to ease passage of stomach contents. An ocular lubricant was applied to the eyes to keep them moist. After clipping of the fleece, the abdomen of the ewe was double scrubbed with a skin disinfectant. Sterile coupling gel was applied to the abdomen and ultrasound examination was used to confirm the gestational age of the ewe^34^ and to assess fetal lie. At the end of the surgery the animal was humanely killed using an overdose of thiopental sodium (40 mg kg^-1^ intravenously).

The practitioner (A.L.D.) identified the umbilical cord as a target. A needle was inserted into the uterine cavity, and the tip was tracked along a trajectory that attained an out-of-plane distance of 15 mm and a depth of 38 mm (Figure 3). Golay coding improved the SNR, with a 7.5-fold increase relative to conventional bipolar excitation (Figure 3B). The 3D tracked needle tip positions were overlaid on the 2D ultrasound image using crosses with widths indicative of the out-of-plane distance and colors indicative of the imaging (step 2.6) (Figure 3C).

**Figure:**
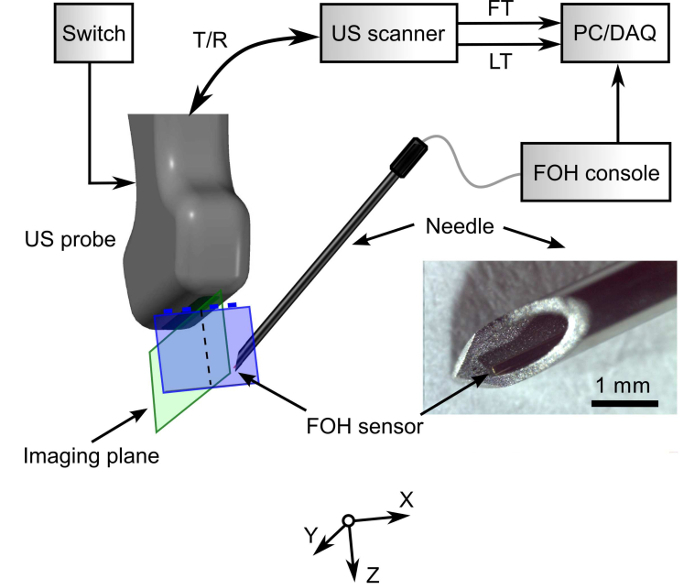

**Figure 1: System overview.** An ultrasound (US) imaging/tracking probe allows for both 2D US imaging and 3D needle tracking. It is driven by an US scanner that provides control over the tracking element transmissions. A switch allows for the electronic selection of transducer elements to alternate between two operation modes: imaging with the central array and tracking with side arrays. A fiber optic hydrophone (FOH) ultrasound receiver, positioned within the lumen of a 20G needle, receives transmissions from the side arrays. T/R: transmit/receive; LT: line trigger; FT: frame trigger; PC: personal computer; DAQ: data acquisition card. This figure and caption are reproduced with permission from Xia, W. *et al.*^14^. Please click here to view a larger version of this figure.

**Figure:**
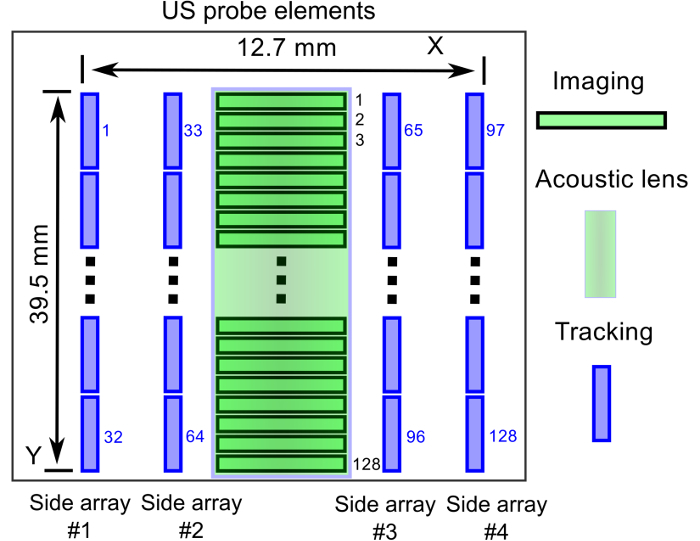

**Figure 2: Transducer element layout of the custom ultrasound imaging probe. **A central array with 128 elements and an acoustic lens enables US imaging. Side arrays, with 32 elements per row and 128 elements in total, enable 3D needle tracking. This figure and caption are reproduced with permission from Xia, W. *et al.*^14^. Please click here to view a larger version of this figure.

**Figure:**
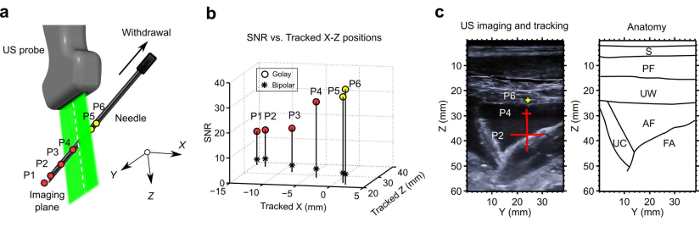

**Figure 3: Needle insertion with 3D tracking *in vivo*. **(**A**) Tracked needle tip positions (circles: P1-P6) obtained during an insertion into the uterine cavity of a pregnant sheep. (**B**) Signal-to-noise ratios (SNRs) of the tracking signals (imaging plane: X = 0). (**C**) Overlay of 3 of the tracked positions onto a 2D US image that was acquired with the central array. The end-to-end length of each cross corresponded to the out-of-plane distance; the color (red/yellow) corresponded to the side of the imaging plane. Key anatomical features are depicted with outlines (right). S: skin; PF: percutaneous fat; UW: uterus wall; AF: amniotic fluid; UC: umbilical cord; FA: fetal abdomen. This figure and caption are reproduced with permission from Xia, W. *et al.*^14^. Please click here to view a larger version of this figure.

## Discussion

Here we demonstrate how 3D ultrasonic tracking can be performed with a custom ultrasound imaging probe and a fiber-optic hydrophone integrated within a needle. From a clinical translation standpoint, several aspects of the custom probe developed in this study are attractive. Its compact size is well-suited for use in small spaces such as the axilla where maneuvering bulky 3D imaging probes is challenging. One limitation of the implementation of the 3D ultrasonic tracking presented here is that manual switching was required to alternate between imaging and tracking modes. In future implementations, this switching could be done directly by the ultrasound imaging system.

The fiber-optic hydrophone is well suited to ultrasonic needle tracking. Its high degree of miniaturization and flexibility allow for its integration into medical devices with small lateral dimensions. Its broad frequency bandwidth^16^ allows for compatibility with different clinical ultrasound probes. Additionally, its omnidirectionality^16^ allows for tracking needles that are inserted at a wide range of angles. Finally, its immunity to disturbances from EM fields and metal objects makes it more suitable to clinical settings in contrast to EM tracking. To achieve greater ultrasound detection sensitivity, a plano-concave Fabry-Pérot cavity could be used in the future^17^. Ultimately, ultrasonic tracking could be combined with other modalities in a single optical fiber, such as reflectance spectroscopy^18^,^19^,^20^,^21^,^22^,^23^, Raman spectroscopy^24^, optical coherence tomography^25^,^26^, and photoacoustic imaging^27^,^28^,^29^,^30^,^31^,^32^,^33^.

Ultrasonic tracking has limitations that are shared with ultrasound imaging. First, tissue heterogeneities will negatively impact ultrasonic tracking; spatial variations in the speed of sound of tissue will decrease the tracking accuracy, as demonstrated by numerical simulations in a previous study^14^. Second, anatomical structures that are highly reflective to ultrasound waves, such as bony structures or air cavities, are probably not compatible with ultrasonic tracking. In future studies, the needle tip position obtained with other imaging modalities, such as 3D rotational C-arm computed X-ray tomography, could be used to assess the accuracy of 3D ultrasonic tracking in heterogeneous tissues *in vivo*.

Despite recent advancements in ultrasound imaging, precise tracking and efficient manipulation of medical devices under the guidance of this modality remain challenging, even for expert practitioners. Active communication between external ultrasound probes and medical devices, as shown here, could improve procedural safety and efficiency. These improvements could greatly facilitate adoption of ultrasound imaging in place of X-ray fluoroscopy in several clinical contexts, such as spinal insertions for interventional pain management. The system developed in this study enables 3D ultrasonic tracking and 2D ultrasound imaging with a compact ultrasound probe. It could improve ultrasound-guided minimally invasive procedures by providing precise localization of the needle tip within current clinical workflow.

## Disclosures

The authors declare that there are no conflicts of interest.
